# Influence of Shoe Mass on Performance and Running Economy in Trained Runners

**DOI:** 10.3389/fphys.2020.573660

**Published:** 2020-09-23

**Authors:** Víctor Rodrigo-Carranza, Fernando González-Mohíno, Jordan Santos-Concejero, Jose Maria González-Ravé

**Affiliations:** ^1^Sport Training Lab, University of Castilla-La Mancha, Toledo, Spain; ^2^Facultad de Lenguas y Educación, Universidad Nebrija, Madrid, Spain; ^3^Department of Physical Education and Sport, University of the Basque Country UPV/EHU, Vitoria-Gasteiz, Spain

**Keywords:** footwear, endurance, oxygen cost, energy cost, athletes

## Abstract

**Purpose:**

The aim of this study was to assess the effects of adding shoe mass on running economy (RE), gait characteristics, neuromuscular variables and performance in a group of trained runners.

**Methods:**

Eleven trained runners (6 men and 5 women) completed four evaluation sessions separated by at least 7 days. The first session consisted of a maximal incremental test where the second ventilatory threshold (VT_2_) and the speed associated to the VO_2max_ (vVO_2max_) were calculated. In the next sessions, RE at 75, 85, and 95% of the VT_2_ and the time to exhaustion (TTE) at vVO_2max_ were assessed in three different shoe mass conditions (control, +50 g and +100 g) in a randomized, counterbalanced crossover design. Biomechanical and neuromuscular variables, blood lactate and energy expenditure were measured during the TTE test.

**Results:**

RE worsened with the increment of shoe mass (Control vs. 100 g) at 85% (7.40%, 4.409 ± 0.29 and 4.735 ± 0.27 kJ⋅kg^−1^⋅km^−1^, *p* = 0.021) and 95% (10.21%, 4.298 ± 0.24 and 4.737 ± 0.45 kJ⋅kg^−1^⋅km^−1^, *p* = 0.005) of VT_2_. HR significantly increased with the addition of mass (50 g) at 75% of VT_2_ (*p* = 0.01) and at 75, 85, and 95% of VT_2_ (*p* = 0.035, 0.03, and 0.03, respectively) with the addition of 100 g. TTE was significantly longer (∼22%, ∼42 s, *p* = 0.002, *ES* = 0.149) in the Control condition vs. 100 g condition, but not between Control vs. 50 g (∼24 s, *p* = 0.094, *ES* = 0.068).

**Conclusion:**

Overall, our findings suggest that adding 100 g per shoe impairs running economy and performance in trained runners without changes in gait characteristics or neuromuscular variables. These findings further support the use of light footwear to optimize running performance.

## Introduction

Running economy (RE) is a key factor that influences long-distance running performance (Conley and Krahenbuhl, 1980) and is usually defined as the steady-state oxygen uptake (VO_2_) required at a given submaximal speed or as the energy requirement per unit of distance run (Fletcher et al., 2009). RE is influenced by multiple factors, including metabolic, cardiorespiratory, neuromuscular, biomechanical, training and environmental factors (Saunders et al., 2004). Some of these factors can be changed chronically through training (Barnes and Kilding, 2015), whereas others can be modified acutely through interventions such as changes in footwear (Hoogkamer et al., 2018). The effects of footwear on running performance is an area of increasing interest (Fuller et al., 2015), especially after the recent sub-2-hour marathon attempts in which footwear played a fundamental role (Hoogkamer et al., 2019; Hunter et al., 2019). If specific shoes can decrease the energy cost of running, athletes would be able to display faster running speeds at a given metabolic rate (Daniels, 1985), which is key when trying to break the marathon world record (Hoogkamer et al., 2018).

One of the main variables related to RE improvements and performance is the shoe mass (Franz et al., 2012; Fuller et al., 2015; Hoogkamer et al., 2016). Previous studies have shown an increase of ∼1% in the energy cost per 100 g of added mass per shoe (Frederick, 1984; Fuller et al., 2015) and a performance reduction of 2% during a 5-km time trial on a treadmill (TT) (Fuller et al., 2016) and 0.78% during a 3-km TT (Hoogkamer et al., 2016). Kipp et al. (2019) predicted slightly smaller improvements at faster running speeds for a given improvement in RE due to the non-linear relationship between oxygen uptake and speed. Therefore, it is necessary to address the effects of shoe mass on RE and performance at higher intensities as the effects at submaximal running speeds cannot be fully extrapolated.

Traditionally, the vVO_2_max (the speed achieved when reaching the maximal oxygen uptake) has been used to evaluate running performance (Billat et al., 1995; Hayes et al., 2004). However, the influence of shoe mass on performance has not been yet studied in a vVO_2_max test to exhaustion.

Previous research has also reported changes in stride length and flight time when extra mass was added on the ankles compared to the lower limbs (Myers and Steudel, 1985). The question arises whether adding mass to the shoes would lead to kinematic changes impairing RE and performance. Secondly, kinematic changes can affect neuromuscular variables (Morin et al., 2007), and stiffness in the lower extremity during running can be influenced by the footwear (Bishop et al., 2006). To the best of our knowledge, there is no research either analyzing the influence of shoe mass on neuromuscular factors and leg and vertical stiffness during high intensity exercise.

Therefore, the aim of the study was to analyze the influence of adding extra shoe mass on RE, time trial running performance at intensity of vVO_2max_, gait characteristics and neuromuscular parameters. We hypothesized that shoe mass would impair high intensity time trial running performance (vVO_2max_) and RE with biomechanical and neuromuscular changes.

## Materials and Methods

### Subjects

Six men (mean ± SD: 20.64 ± 1.60 years; 60.70 ± 6.91 kg and 170.83 ± 6.49 cm) and five women (mean ± SD: 22.12 ± 1.03 years; 51.62 ± 9.58 kg and 161.20 ± 6.22 cm) trained runners volunteered to participate in this study. All participants were experienced middle- and long-distance runners and were free from injury for 6 months prior to testing. The participants had a 10-km race time that ranged between 32 and 34 min and they had participated in several cross-country and middle- and long-distance National Championships in sub20 and sub23 category. Prior to the study, all participants were informed about the testing protocols, possible risks involved and were invited to provide written informed consent. The study was performed in accordance with the principles of the Declaration of Helsinki (October 2008, Seoul), and the experimental protocols were approved by the local ethics committee.

### Experimental Design

The effect of shoe mass on RE and time to exhaustion (TTE) was evaluated using a randomized counterbalanced experimental design. All participants visited the laboratory on four different occasions separated by at least 7 days in a non-fatigued state (no intense exercise in the previous 48 h). All testing sessions were performed in the same laboratory under similar environmental conditions (550 m altitude, 20–25°C, 35–40% relative humidity). All runners followed a similar pre-competition diet 24 h before testing, which was performed at the same hour of the day to avoid any influence of circadian rhythms. Participants used their preferred racing shoes throughout the study. The shoe mass varied between 178 and 247 g for size 35–44 EU. The shoe mass for the control condition was controlled, because each runner used their own shoe for the experimental conditions (+50 and +100 g).

During the first visit, anthropometrical variables were measured. Height was measured to the nearest 0.1 cm with a portable stadiometer (Seca, Bonn, Germany) and body mass was measured to the nearest 0.1 kg with a portable balance (Seca, Bonn, Germany), whilst barefoot and wearing light shorts. Then, all runners completed an incremental maximal running test on a treadmill (HP Cosmos Pulsar, H/P/Cosmos Sports & Medical GMBH, Nussdorf-Traunstein, Germany). The test started at 2.50 m⋅s^−1^ for 5-minute (warm-up). Then, the speed increased by 0.28 m⋅s^−1^ every minute until volitional exhaustion. The treadmill slope was kept at 1% to imitate external air resistance (Jones and Doust, 1996). During the test, respiratory variables were measured using a gas analyzer (CPX Ultima Series MedGraphics, St. Paul, MI, United States), which was calibrated prior to each session (CO_2_ 4.10%; O_2_ 15.92%). The zirconia O_2_ analyzer has a response time of <0.80 ms and accuracy of ±0.03% and a CO_2_ analyzer a response time of <130 ms and accuracy of ±0.10%. The average of VO_2_ value obtained during the last 30s of the final running stage was considered as VO_2_max when as least two of the following criteria were fulfilled (Howley et al., 1995): (1) a *plateau* in VO_2_ (an increase of less than 1.5 ml⋅kg^−1^⋅min^−1^ in two consecutive workloads; (2) Respiratory exchange ratio (RER) >1.15; (3) maximal HR values above 95% of the age-predicted maximum (220-age). The minimal speed needed to elicit VO_2_max was considered as vVO_2_max (Billat et al., 1996). The second ventilatory threshold (VT_2_) was identified by the non-linear increase in VE/VCO_2_ curve concomitant to a second strong increase in VE/VO_2_, with a further increase in exercise intensity (Reinhard et al., 1979). The intensity corresponding with the VT_2_ was used to establish the intensity for RE assessments.

**FIGURE 1:**
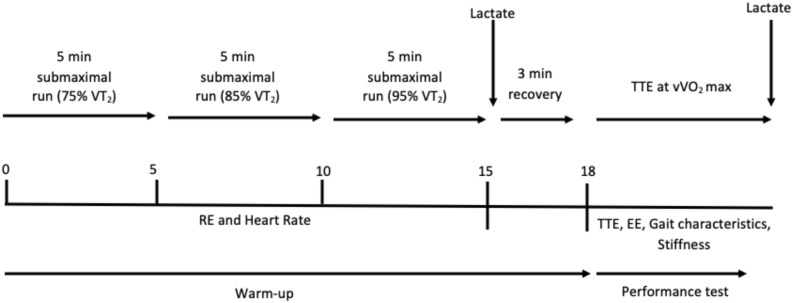
Overview of cross-over experimental design. Running economy (RE), TTE (Time to exhaustion), energy expenditure (EE), Second ventilatory threshold (VT_2_) and the speed associated to the VO_2max_ (vVO_2max_).

**FIGURE 2:**
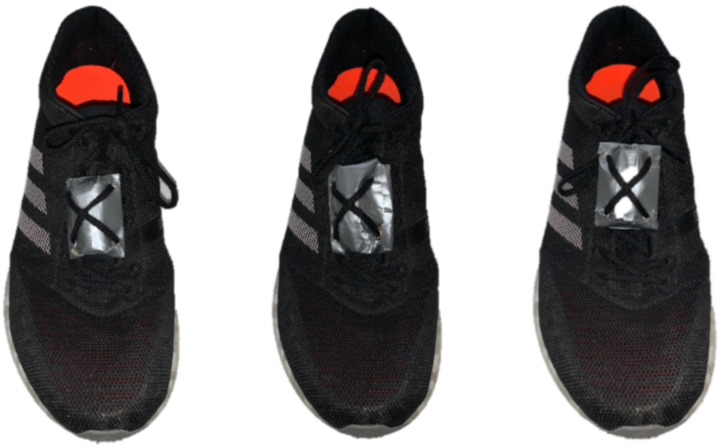
Shoe mass conditions. From left to right: Control (4 g), 50 and 100 g.

During the second, third and fourth session, participants completed three TTE tests at the speed of VO_2_max (in a cross-over experimental design), preceded by three different warm-up conditions (different shoe mass) in a randomized order and on separate days. The TTE tests were performed with the shoe mass used during the warm-up (Figure 1) in order to evaluate the influence of increase shoe mass on the performance at high intensities. The warm-up consisted of 15-minute (3 × 5-minute) at intensities corresponding to the 75, 85, and 95% of the VT_2_ (1% gradient) without recovery, in order to assess RE and HR. Before the warm-up (during the 3 min recovery after the warm-up), the researchers put on the subject’s shoes a platen (4 g) and added 50 or 100 g (experimental conditions) of lead pellets per shoe in order to manipulate the mass of the shoes, similar to a timing chip worn on the shoelace. Participants did not manipulate their shoes and were shod by a researcher, keeping them unaware of the aim of study and the extra-mass used as treatment. The platen alone was considered as Control condition (4 g) (Figure 2).

Running economy at each speed (75, 85, and 95% of VT_2_) was expressed as oxygen cost per time (ml⋅kg^−1^⋅min^−1^), oxygen cost per distance (ml⋅kg^−1^⋅km^−1^) and energy cost per distance (kJ⋅kg^−1^⋅km^−1^) in order to take into account the substrate use and was calculated as detailed elsewhere (González-Mohíno et al., 2018). In order to verify whether a steady-state of VO2 had been achieved, the difference between the fourth and fifth minute of each 5-minute period at 75, 85, and 95% of the VT2 was calculated. A difference smaller than the minimal detectable change (MDC) was used to confirm a *plateau* had been achieved, following the previous study of Blagrove et al. (2017). For example, the difference in the control condition at 95% of VT_2_ was 0.22 ml⋅kg^−1^⋅min^−1^. The MDC was 1.74 ml⋅kg^−1^⋅min^−1^, thus, the *plateau* was achieved in all participants. Another example in the 100 g condition at 95% of VT_2_, the difference was 0.2 ml⋅kg^−1^⋅min^−1^ and the MDC was 1.7 ml⋅kg^−1^⋅min^−1^. With the average of RER over the last 2 min at each submaximal intensity (75% of VT_2_ = 0.82 ± 0.05, 0.85 ± 0.05, 0.86 ± 0.06; 85% of VT_2_ = 0.92 ± 0.07, 0.95 ± 0.07, 0.97 ± 0.05 and 95% of VT_2_ = 0.96 ± 0.08, 0.98 ± 0.07, 0.99 ± 0.05, for control, 50 and 100 g conditions, respectively), the caloric equivalent of the VO_2_ (kcal/l O_2_) was determined (Lusk, 1928) and the energy cost was calculated (kJ⋅kg^−1^⋅km^−1^). The equation used was the following:

VO_2_ ⋅ caloric equivalent ⋅ s^−1^⋅ BM^−1^⋅ K, where VO_2_ is measured in liters per minute, caloric equivalent is in kilojoules per liter, speed (s) is in meters per minute, body mass (BM) is in kilograms, and K is 1,000 m.

Blood samples (0.5 μl) were collected from the fingertip in the 30 s before starting the TTE and 30 s after the end of the Time to exhaustion tests for blood lactate determination (Lactate Scout, SensLab GmbH, Germany). Spatiotemporal parameters of the gait cycle [contact time (CT), step frequency (SF), stride length (SL), and flight time (FT)] were measured for every step during treadmill running using an optical measurement system (Optojump-next, Microgate, Bolzano, Italy) during all the entire TTE tests, with a sampling frequency of 1000Hz. Data were recorded and 1 central minute of the TTE test was averaged for subsequent analyses. Neuromuscular variables (leg and vertical stiffness) were determined according to Morin et al. (2005). Vertical stiffness (Kvert) was calculated as the ratio of the center of mass. Leg stiffness (Kleg) was calculated as the ratio of peak vertical force to the maximum leg spring compression. Kleg and Kvert were measured during all the entire TTE tests.

Lastly, the energy expenditure (kJ) was calculated using the value of oxygen uptake (ml min^−1^) and the blood lactate concentration [(La^–^)] (mmol⋅l^−1^) during the TTE test using the GEDAE-LaB software by Bertuzzi et al. (2016) previously used in other study (González-Mohíno et al., 2018). A value of 1 mmol⋅l^−1^ is considered to be equivalent to 3 ml O_2_⋅kg^−1^ body mass. The energy expenditure and energy systems were expressed in kilojoules assuming caloric equivalents of 20.90 kJ⋅l O${}_{2}^{-1}$ (Di Prampero and Ferretti, 1999).

### Statistical Analysis

Statistical analyses were conducted using JASP software (version 0.11.1.0) for Mac. Data were screened for normality of distribution and homogeneity of variance using a Shapiro-Wilk Normality Test. Repeated-measures ANOVA was conducted to compare shoe mass conditions. When a significant main effect for shoe was observed, a Bonferroni *post hoc* test was performed. A two-way repeated-measures ANOVA was performed to analyze the effect of the interaction sex-condition. ANCOVA was conducted to analyze the effects of shoe mass on EC with body mass as the covariate, following the previous study of Davies et al. (1997). The effect size was calculated using the partial eta squared (η2) in the repeated-measures ANOVA and ANCOVA. Values of 0.01, 0.06, and above 0.15 were considered as small, medium and large, respectively (Cohen, 1992). Significance level for all analyses was set at α = 0.05.

## Results

Descriptive characteristics of the participants in the incremental running test are shown in the Table 1.

**TABLE 1:** Descriptive data of the incremental maximal running test.

	Men	Women
VO_2_max (ml⋅kg^−1^⋅min^−1^)	70.20 ± 3.66	60.14 ± 6.19
vVO_2_max (km⋅h^−1^)	18.84 ± 1.84	17.41 ± 1.34
RERmax	1.26 ± 0.07	1.25 ± 0.06
HRmax (bpm)	197.33 ± 6.05	198.21 ± 5.31
vVT_2_ (km⋅h^−1^)	16.60 ± 1.67	14.00 ± 0.70
VT_2_ (%)	78.93 ± 8.24	81.51 ± 4.09
75% VT_2_ (km⋅h^−1^)	11.86 ± 1.73	10.50 ± 1.13
85% VT_2_ (km⋅h^−1^)	13.46 ± 1.96	11.91 ± 1.28
95% VT_2_ (km⋅h^−1^)	15.38 ± 1.72	13.31 ± 1.44

*VT_2_, second ventilatory threshold; RER, Respiratory exchange ratio; HR, Heart rate.*

Table 2 depicts the results of the submaximal variables and performance test variables during the study for each shoe condition. No significant differences were found in any submaximal variable between sexes in relation to the changes between shoe mass conditions (condition × sex interaction).

**TABLE 2:** Results for the variables analyzed during the study for each shoe condition.

	Conditions			Repeated measures ANOVA (condition)		Repeated measures ANOVA (condition x sex)	
	Control	50 g	100 g	*p*	η^2^	*p*	η^2^
**Performance test**							
TTE (s)	193.40 ± 40.67	169.30 ± 49.63	151.20 ± 39.60^#^**	<0.001	0.149	0.151	0.023
SL (cm)	308.81 ± 35.31	314.20 ± 25.71	318.11 ± 33.25	0.097	0.016	0.366	0.006
SF (step/min)	197.13 ± 11.91	201.28 ± 48.15	193.90 ± 14.45	0.779	0.011	0.355	0.049
CT (s)	0.199 ± 0.02	0.198 ± 0.02	0.196 ± 0.02	0.642	0.004	0.162	0.016
FT (s)	0.111 ± 0.02	0.113 ± 0.01	0.117 ± 0.02	0.472	0.018	0.72	0.008
Kleg (kN⋅m^−1^)	7.11 ± 1.87	7.06 ± 1.39	7.49 ± 1.87	0.387	0.014	0.389	0.014
Kvert (kN⋅m^−1^)	30.82 ± 8.25	30.69 ± 7.08	32.46 ± 8.86	0.405	0.011	0.354	0.012
Aerobic (%)	85.20 ± 5.76	83.79 ± 4.55	82.41 ± 4.07	0.164	0.058	0.746	0.009
Anaerobic (%)	14.80 ± 5.76	16.21 ± 4.54	17.59 ± 4.07	0.164	0.058	0.746	0.009
EE aerobic (kJ)	45.15 ± 18.68	41.78 ± 12.28	41.51 ± 10.89	0.588	0.015	0.517	0.019
EE anaerobic (kJ)	7.31 ± 2.47	7.62 ± 1.35	8.52 ± 1.48	0.082	0.080	0.255	0.039
EE total (kJ)	52.45 ± 19.36	49.40 ± 12.47	50.03 ± 11.29	0.702	0.009	0.381	0.025
[La^–^] pre (mmol l^−1^)	3.35 ± 1.14	4.61 ± 2.12	5.88 ± 3.31^#^*	0.005	0.174	0.527	0.016
[La^–^] post (mmol l^−1^)	11.63 ± 2.04	12.18 ± 1.34	13.61 ± 1.56^†∗#∗^	0.01	0.217	0.274	0.048
Δ[La^–^] (mmol l^−1^)	8.52 ± 2.48	9.59 ± 2.13	9.00 ± 1.05	0.40	0.051	0.367	0.058
**Submaximal variables**							
HR 75% VT_2_ (bpm)	155.30 ± 13.2	159.90 ± 14.38^#^**	163.30 ± 14.70^#^*	0.008	0.057	0.737	0.003
HR 85% VT_2_ (bpm)	170.50 ± 11.42	173.40 ± 9.30	176.40 ± 12.79^#^*	0.012	0.048	0.333	0.01
HR 95% VT_2_ (bpm)	182.71 ± 10.20	185.40 ± 8.58	186.50 ± 10.29^#^*	<0.011	0.029	0.222	0.008

Data are presented as mean ± standard deviation. TTE, Time to exhaustion; SL, stride length; SF, step frequency; CT, contact time; FT, flight time; Kleg, leg stiffness; Kvert, vertical stiffness; EE, energy expenditure; [La^–^], blood lactate; HR, heart rate; *Post hoc* analysis: # Different from Control; different from 50 g ^†^; *p < 0.05; **p < 0.01.

### Submaximal Variables

**FIGURE 3:**
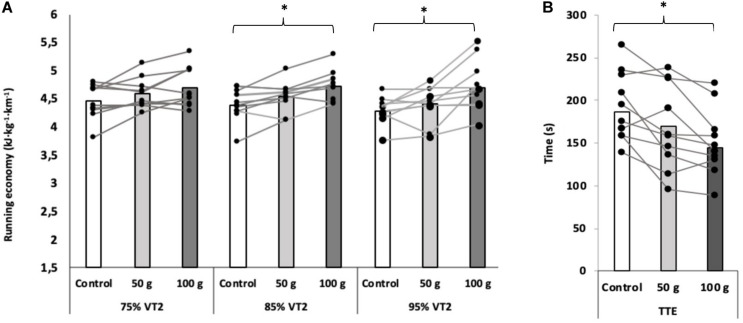
Running economy (kJ⋅kg^−1^⋅km^–1^) at 75, 85, and 95% of the VT_2_ **(A)** and TTE test performance **(B)**. Bar graphs represent mean values, circles joined by lines represent runners. *p ≤ 0.05 during *post hoc* comparisons when main effect of footwear significant. *n* = 11.

Running economy (expressed as energy cost) significantly worsened with the addition of shoe mass (Control vs. 100 g) at 85% of VT_2_ (4.41 ± 0.29 and 4.73 ± 0.27 kJ⋅kg^−1^⋅km^−1^, *p* = 0.021) and 95% (4.30 ± 0.24 and 4.74 ± 0.45 kJ⋅kg^−1^⋅km^−1^, *p* = 0.005) but there were no differences between 50 vs. 100 g nor between Control vs. 50 g at any intensity (Table 3 and Figure 3). RE (expressed as oxygen cost per distance) significantly worsened with the addition of shoe mass (Control vs. 100 g) at 85% of VT_2_ (214.16 ± 11.54 and 225.17 ± 13.02 ml⋅kg^−1^⋅km^−1^, *p* = 0.008). However, when RE was expressed as oxygen cost per time, there were no significant differences between conditions. Results of the ANCOVA test revealed that RE did not differ between men and women. HR significantly increased with the addition of 50 g at 75% of VT_2_ compared to Control. At the intensities of 75, 85, and 95% of VT_2_ there was an increase of HR in the 100 g condition compared to Control (*p* = 0.03), with no differences between 50 vs. 100 g at any intensity (Table 1).

**TABLE 3:** Results of the RE for each shoe condition (body mass adjustment).

	Conditions			ANCOVA (condition)		ANCOVA (condition × sex)	
	Control	50 g	100 g	*p*	η^2^	*p*	η^2^
**Submaximal variables**							
RE 75% VT_2_ (kJ⋅kg^−1^⋅km^−1^)	4.46 ± 0.30	4.59 ± 0.27	4.71 ± 0.36	0.091	0.175	0.696	0.016
RE 85% VT_2_ (kJ⋅kg^−1^⋅km^−1^)	4.41 ± 0.29	4.53 ± 0.27	4.73 ± 0.27^†∗#∗^	0.009	0.314	0.389	0.029
RE 95% VT_2_ (kJ⋅kg^−1^⋅km^−1^)	4.30 ± 0.24	4.40 ± 0.32	4.74 ± 0.45^†∗#∗∗^	0.012	0.296	0.676	0.031
RE 75% VT_2_ (ml⋅kg^−1^⋅min^−1^)	42.99 ± 5.76	43.07 ± 4.75	44.22 ± 4.87	0.586	0.042	0.560	0.045
RE 85% VT_2_ (ml⋅kg^−1^⋅min^−1^)	47.19 ± 5.68	47.36 ± 4.63	49.77 ± 5.24	0.159	0.137	0.530	0.050
RE 95% VT_2_ (ml⋅kg^−1^⋅min^−1^)	51.27 ± 5.89	52.28 ± 5.25	54.04 ± 5.77	0.189	0.125	0.523	0.050
RE 75% VT_2_ (ml⋅kg^−1^⋅km^−1^)	222.04 ± 12.43	223.79 ± 17.13	229.92 ± 13.89	0.039	0.160	0.741	0.024
RE 85% VT_2_ (ml⋅kg^−1^⋅km^−1^)	214.16 ± 11.54	217.44 ± 15.91	225.17 ± 13.02^#^*	0.006	0.269	0.804	0.060
RE 95% VT_2_ (ml⋅kg^−1^⋅km^−1^)	205.50 ± 10.87	213.50 ± 16.71	217.13 ± 15.67	0.373	0.032	0.123	0.884

Data are presented as mean ± standard deviation. RE, running economy; *Post hoc* analysis: # Different from Control; different from 50 g †; *p < 0.05; **p < 0.01.

### Performance Test

Time to exhaustion was longer (∼42 s, *p* = 0.002, *ES* = 1.58) in Control vs. 100 g condition, being affected by the mass added. However, there were not significant differences between Control vs. 50 g condition (∼24s, *p* = 0.094, *ES* = 0.80), neither between 50 vs. 100g condition (∼18 s, *p* = 0.108, *ES* = 0.78) (Figure 3B).

There were significant (*p* = 0.002) changes in [La^–^] between Control vs. 100 g condition at the end of the TTE test (11.63 ± 2.04 mmol l^−1^ vs. 13.61 ± 1.56 mmol l^−1^) and between 50 vs. 100 g (12.18 ± 1.34 vs. 13.61 ± 1.56 mmol l^−1^) but not between Control vs. 50 g. Before the TTE test, the 100 g condition displayed higher [La^–^] vs. Control (5.88 ± 3.31 vs. 3.35 ± 1.14 mmol l^−1^, *p* = 0.038). There were no significant differences in Δ[La^–^] in the TTE test (*p* = 0.40) between conditions (8.28 ± 1.50, 7.57 ± 1.73, and 7.73 ± 2.43 mmol l^−1^ for Control, 50 and 100 g, respectively).

There were no significant differences between shoe mass conditions for the total energy expenditure, energy system contributions (aerobic and anaerobic) during the TTE test. However, the anaerobic expenditure (kJ) showed a positive trend (no significant differences, *p* = 0.082) with the addition of shoe mass (4.10 and 14.20% for 50 and 100 g, respectively) with small-moderate effect sizes (*ES* = 0.058 and 0.072 for 50 and 100 g, respectively) compared to Control.

Regarding the spatiotemporal parameters and neuromuscular stiffness (Kvert and Kleg), we found no significant differences between shoe mass conditions during the TTE test (*p* > 0.05).

## Discussion

The aim of this study was to assess the effects of adding extra shoe mass (50 and 100 g) on RE, HR, gait characteristics, neuromuscular variables and performance in trained runners. The main finding was a reduction of performance (∼22%) in a TTE test at constant speed corresponding to vVO_2_max when 100 g were added per shoe. In addition, we found an increase in the energy cost of running (worse RE) when 100g were added per shoe at 85 and 95% of VT_2_ (7.40 and 10.20%, respectively), although there were no differences when 50g were added.

Our results are in accordance with a previous meta-analysis (Fuller et al., 2015) where a positive association between shoe mass and the metabolic cost of running was found. Previous studies (Frederick, 1984; Franz et al., 2012; Hoogkamer et al., 2016) have reported an increment in the oxygen cost of ∼1% per added 100 g of shoe mass, although in the current study, RE was worsened to a greater extent (7.40 and 10.21 at 85 and 95% of VT_2_, respectively). The greater deterioration observed in the current study when compared to the literature may be due to the fact that this is the only one in which participants ran with a 1% slope. Also, previous studies (Frederick, 1984; Franz et al., 2012; Hoogkamer et al., 2016) used a set speed for all the runners instead of a relative intensity. Therefore, the intensity could be different between the runners. Other factors, including having RE expressed also as energy cost instead of just oxygen cost as in other studies could also have affected the results. In our study, there were no significant differences between conditions when RE was expressed as oxygen cost. Therefore, as Fletcher et al. (2009) suggested previously, the expression of RE as energy cost seems more sensitive to changes in speed than the oxygen cost. In addition, there were no differences between the sexes, which shows that the increase in shoe mass affects men and women equally. The RE values of the participants in the current study are lower than those reported in previous studies for runners of similar athletic ability. Williams et al. (1991) found oxygen cost values ranging between 197 and 200 ml⋅kg^−1^⋅km^−1^ at 11–13 km⋅h^−1^ in moderately trained runners, whereas runners in the current study presented values of 214–222 ml⋅kg^−1^⋅km^−1^ at similar intensities, despite having better VO_2_max levels (60–70 vs. 55–66 ml⋅kg^−1^⋅min^−1^, respectively). This may be explained for the inverse relationship between RE and VO_2_max (Morgan and Daniels, 1994; Morgan et al., 1995; and Patte et al., 1995), were high VO_2_max values can compensate for a relatively poor RE.

These RE differences reported may only be relevant for long-distance races where the pace is similar to the submaximal intensities used to measure RE. Previous studies (Fletcher et al., 2009; Shaw et al., 2014) found a linear increase in RE (energy cost) with speed, being more sensitive to changes in speed. However, a recent study reported a non-linear relationship between RE (oxygen cost) and running speed (Kipp et al., 2019). The authors concluded that the improvement in speed is slightly greater than the relative improvement in RE. Thus, we cannot ensure that the changes observed at these submaximal intensities would be translated to changes in running performance at higher intensities (Batliner et al., 2018). That is why this study was the first to investigate the effects of adding extra shoe mass on running performance during a time to exhaustion test at vVO_2_max. In agreement with Frederick (1984), who suggested that the effects of adding shoe mass on submaximal intensities are dependent on running speed, we found a linear impairment of RE at 75, 85, and 95% of VT_2_ (5.51, 7.40, and 10.21%, respectively). However, if performance changes at higher intensities were directly proportional to RE changes at lower intensities, the expected reduction should be ∼15%, and not the ∼22% impairment we found when 100 g were added per shoe. This means that other factors may have also affected running performance in this study.

During the TTE test, total energy expenditure (kJ) remained unchanged, although the anaerobic energy expenditure showed a trend to increase with the added mass (*p* = 0.082; η^2^ = 0.08). This trend in the anaerobic metabolism may be due to significant differences in [La^–^] after the TTE between conditions. [La^–^] is sensitive to changes in exercise intensity and duration (Beneke et al., 2011) being in the current study influenced by the increase in the shoe mass. The [La^–^] increase observed in this study may have affected RE (Hoff et al., 2016) and TTE performance (Midgley et al., 2006). In addition, just before the TTE, [La^–^] was higher in the 100 g condition when compared to control and 50 g. This could be due to the influence of added shoe mass during warm-up, which may cause more fatigue and, therefore a longer recovery should have been used between the warm-up and the TTE test.

Regarding the kinematic results of our study, these data are in line with previous research. Minimalist shoes (Fuller et al., 2016) and heavier shoes (Becker and Borgia, 2020) have small acute effects on stride length and frequency compared to conventional shoes. In our study, we found non-significant stride length changes associated to the added mass (*p* = 0.097; η^2^ = 0.016). Step frequency, contact time and flight time remained unchanged in our study. Gazeau et al. (1997) found a significant increase in stride length and contact time at vVO_2_max intensity, with no significant increments in flight time. However, Hayes et al. (2004) found that stride length, contact time and flight remained unchanged. In relation to the neuromuscular variables, we found no significant changes in the neuromuscular variables between shoe mass conditions, similar to the results of Divert et al. (2008). The differences in leg stiffness are primarily due to reduced contact time (Morin et al., 2007) produced by increased ground impact forces (Pollard et al., 2018) and muscle activity (Becker and Borgia, 2020). A possible explanation for the lack of changes in Kleg and Kvert could be that there were no contact time differences between shoe conditions (*p* = 0.642; η^2^ = 0.004). Therefore, this study showed that the addition of 100 g per shoe had no influence on the kinematic and neuromuscular variables that could explain the changes in performance.

Lastly, we found that HR increased with the running speed and the addition of shoe mass. It is well known that there is a linear relationship between HR, exercise intensity and oxygen consumption (Arts and Kuipers, 1994). Thus, if RE is determined by measuring the steady-state oxygen uptake and respiratory exchange ratios during running (Saunders et al., 2004), it is reasonable to think that HR will increase in a similar proportion when compared to the energy cost of running. This higher HR may just be translated as a higher effort consequence of the extra shoe mass.

## Limitations

We faced several limitations. For example, Fuller et al. (2016) suggested that apart from shoe mass, other factors not analyzed in the current study, may influence RE (i.e., shoe cushioning). However, previous research reported similar VO_2_ and HR values in runners using shoes with different midsole characteristics but with similar shoe mass (Mitschke et al., 2019), indicating that shoe mass may be the most relevant variable when analyzing the influence of footwear on RE. In addition, the TTE was performed after the submaximal test (warm-up), and therefore, the participants could have accumulated fatigue that may have affected the TTE test performance. For example, the high baseline [La-] values could have affected the final TTE test performance due to the shoe mass added, although runners were given 3 min of recovery to mitigate the effects of fatigue after the submaximal test. The warm-up and recovery time prior to the TTE test should be considered when analyzing the effects of shoe mass on the TTE test performance in future studies.

## Conclusion

These results suggest that shoe mass is a key factor in endurance running performance at vVO_2_max intensity. The effect of shoe mass seems to have more influence on performance at high intensities (vVO_2_max) compared to the RE changes observed at submaximal intensities. On the basis of these results, we recommend choosing the lightest shoes to optimize the performance at high and submaximal intensities.

## Data Availability Statement

The raw data supporting the conclusions of this article will be made available by the authors, without undue reservation.

## Ethics Statement

The studies involving human participants were reviewed and approved by Facultad de Lenguas y Educación, Universidad Nebrija, Madrid, Spain (FGM02102019). The patients/participants provided their written informed consent to participate in this study.

## Author Contributions

All authors made a significant contribution to the final version of this manuscript and contributed to the interpretation of the results. VR-C and FG-M conceived and planned the experiments. VR-C, FG-M, and JG-R carried out the experiments. VR-C, FG-M, and JG-R contributed to the sample preparation. VR-C took the lead in writing the manuscript and it was completed by FG-M, JS-C, and JG-R.

## Conflict of Interest

The authors declare that the research was conducted in the absence of any commercial or financial relationships that could be construed as a potential conflict of interest.
